# In children with attention-deficit/hyperactivity disorder, less task-related up-modulation of motor cortex during response inhibition

**DOI:** 10.1002/cns3.20101

**Published:** 2025-01-15

**Authors:** Donald L. Gilbert, Deana Crocetti, Paul S. Horn, Steve W. Wu, David A. Huddleston, Jacqueline M. Ehrman, Karlee Y. Migneault, Stewart H. Mostofsky

**Affiliations:** 1 Division of Neurology, Cincinnati Children’s Hospital Medical Center, Cincinnati, Ohio, USA; 2 Department of Pediatrics, University of Cincinnati College of Medicine, Cincinnati, Ohio, USA; 3 Center for Neurodevelopmental and Imaging Research, Kennedy Krieger Institute, Baltimore, Maryland, USA; 4 Departments of Neurology and Psychiatry, Johns Hopkins University School of Medicine, Baltimore, Maryland, USA; 5 Medical Scientist Training Program, University of Cincinnati College of Medicine, Cincinnati, Ohio, USA

**Keywords:** attention-deficit/hyperactivity disorder, children, motor cortex, response inhibition, transcranial magnetic stimulation, task-related up-modulation (TRUM)

## Abstract

**Objective:**

The aim of this study was to identify a quantitative, brain-based measure reflecting impaired response inhibition in children with attention-deficit/hyperactivity disorder (ADHD).

**Methods:**

In this cross-sectional study, we used transcranial magnetic stimulation (TMS) to evoke potentials in hand muscle during both a simple reaction time and a response inhibition task in 8-to-12-year-old children, 41 with ADHD (42% girls, 76% white, mean age 10.3 years) and 38 typically developing controls (53% girls, 74% white, mean age 9.8 years). We used mixed-model linear regressions of evoked potential amplitudes to compare motor cortex excitability at (1) task-onset (“START”: 550 ms prior to action); (2) preparing-to-go (“GO”: 150 ms prior to action); and (3) selecting-to-stop (“STOP”: 150 ms after stop cue). We hypothesized that task-related up-modulation of motor cortex excitability (motor evoked potential amplitudes) would depend both on task (STOP > GO > START) and on diagnosis (controls > patients).

**Results:**

Motor cortex up-modulation was significantly greater for STOP trials than during GO or START. Children with ADHD had both worse response inhibition performance (longer stop-signal reaction times) and significantly less task effect on motor cortex up-modulation. The largest diagnostic difference in motor cortex activation occurred during STOP trials. Reduced up-modulation during stopping was also associated with higher parent-rated symptom severity.

**Interpretation:**

Our findings suggest that motor cortex up-modulation of excitability, assessed indirectly by TMS motor evoked potentials, reflects the cognitive load during response inhibition tasks and may be a quantitative, brain-based indicator of impaired response inhibition in children with ADHD.

## Introduction

Attention-deficit/hyperactivity disorder (ADHD), the most common pediatric behavioral diagnosis,^1^ elevates risk for subsequent substance abuse, incarceration, and impulse control disorders in adulthood.^2^ Its cardinal symptoms, impulsivity, hyperactivity, and inattention, are pervasive in many complex disorders, such as epilepsies,^3^ and monogenetic disorders, such as neurofibromatosis.^4^ Developing more effective treatments has been hampered by the reductionist diagnostic process, which classifies ADHD as categorically present or absent solely based on subjective behavioral ratings of symptoms exceeding a population threshold. This approach fails to account for heterogeneity from biological processes that might respond differentially to medical or behavioral treatments. Approaches that incorporate objective, biological measures^5,6^ might complement symptom-based diagnoses and allow for identification of biologically based subtypes.

To identify useful biological measures, we and others have sought to identify brain-based, quantitative biomarkers that reflect domains of impaired function. As children with ADHD manifest impaired motor control,^7,8^ we and others have focused on physiology of motor cortex (M1), using transcranial magnetic stimulation (TMS) to demonstrate that children with ADHD show reduced resting M1 short-interval cortical inhibition (SICI).^9–11^ Moreover, SICI correlates with ADHD symptom severity,^12,13^ M1 GABA levels,^14^ and responses to both stimulant^15^ and nonstimulant^16^ medications. However, there is overlap between SICI in children with ADHD and typically developing (TD) control children. This has supported finding additional TMS-based biomarkers of ADHD that might complement SICI,^17^ reflect other domains of impaired function,^18,19^ and ultimately address biological heterogeneity in ways that can help advance clinical care.^20^

Subsequent research in ADHD has thus sought to quantify M1 physiology during performance of diagnosis-relevant actions. Based in primate studies showing that motor cortex pyramidal cells begin to discharge in anticipation of movement onset,^21,22^ investigators have used TMS to study human M1 physiology prior to self-paced and cued movements^23^ as well as during inhibition of actions.^24^ Our rationale for choosing TMS during motor tasks requiring response inhibition^25^ is based on studies showing that impulsive and hyperactive children manifest impairments in this form of cognitive control.^26–28^

To this end, we recently demonstrated reduced activation of M1 during engagement in a stop-signal task in school-age children with ADHD compared with TD children.^17^ This finding of reduced “task-related up-modulation (TRUM)” in children with ADHD had some limitations. First, in our child-friendly, race-car Slater-Hammel paradigm,^24,29^ behavioral performance did not differ for children with ADHD. Second, specificity of reduced TRUM for response inhibition could not be determined.

Addressing those limitations, the present study in a new, identically phenotyped cohort modifies the experimental protocol to measure TRUM distinctly during initial task engagement, going-without-stopping, and response inhibition conditions. Using this approach, we hypothesized that (1) across all children, TRUM will be largest during stopping; (2) across all tasks, children with ADHD will show lower TRUM; and (3) the effect of the ADHD diagnosis on TRUM will be greatest during stopping. Further, we hypothesized that reduced TRUM would be associated with more severe clinical ADHD rating scale scores and worse response inhibition performance (i.e., longer stop-signal reaction times—SSRTs).

## Materials and Methods

### Participants

This is a cross-sectional study of neurobiological mechanisms underlying response inhibition in 8-to-12-year-old children. Both ADHD and TD control children were recruited through community and hospital-based advertisements in two United States cities from June 2021 to October 2023. Assessment for eligibility was identical to our prior studies,^11,17^ with the exception that we used the semi-structured Kiddie Schedule for Affective Disorders and Schizophrenia for School-Age Children, Present and Lifetime Version (K-SADS-PL DSM5).^30^ Parents completed the ADHD-RS^31^ rating or the Conners ADHD scale.^32^ Review of all electronic health system and research protocol diagnostic information by a board-certified pediatric neurologist with extensive clinical experience with ADHD was required for inclusion and diagnostic classification. Exclusion criteria for TD controls included past or current history of any neurological psychiatric disorders, along with past history of significant head injury, seizures, major medical conditions, prescribed neurological or psychiatric medications, a full-scale IQ <80 on the Wechsler Intelligence Scale for Children V,^33^ or any standard exclusion criteria for TMS (hearing loss, implanted medical devices, etc.).^34^ Identical exclusion criteria were applied for ADHD participants. Participants with ADHD prescribed stimulant medication had to agree to temporarily discontinue these medications the day before and day of study visits. Parents gave written informed consent, and participants ages 11 years and older provided written assent. The Cincinnati Children’s Hospital Medical Center Institutional Review Board approved this as a minimal-risk study with additional approval, under a reliance agreement by Johns Hopkins School of Medicine Institutional Review Board.

### Behavioral Questionnaires—ADHD Symptom Ratings

The DuPaul ADHD Rating Scale (ADHD-RS)^31^ is an 18-item scale used to assess inattentive and hyperactive severity in children and teens ages 6–18 years. Parents ranked how often (0 = *never or rarely* to 3 = *very often*) their child exhibited certain behaviors (e.g., “Is easily distracted”) in the past 6 months. Items coded as 2 or 3 were counted as ADHD symptoms. Raw scores for the sum of all responses as well as inattentive and hyperactive/impulsive domains were used as covariates during data analysis. For diagnosis, we inadvertently administered an alternate, validated scale^32^ to parents of nine participants. For consistency, we only used ADHD-RS scores in regression.

### Response Inhibition: The Slater-Hammel Anticipated Response Inhibition Task, Race-Car Version

The Slater-Hammel task is a self-paced, anticipated response inhibition SSRT task during which individuals execute (GO) or withhold (STOP/inhibit) a planned response, such as lifting a finger when a moving indicator approaches a predefined target. The GO (respond) and STOP (inhibit-response) trials are randomly presented and biased toward GO (e.g., 3:1 GO:STOP). The STOP cue, presented randomly after the onset of the trial, is a salient stimulus after which the participants must inhibit the primed GO response. The Slater-Hammel task creates consistency in timing of action and inhibition, allowing for programming TMS pulses at prespecified times to probe M1 physiology during response inhibition.^24^

#### Dynamic stopping to evaluate SSRT

The timing of the initial STOP cue is moderately difficult, at 500 ms, which is 300 ms prior to the anticipated GO. STOP cues shift based on success (50 ms later, more difficult) or failure (50 ms earlier, less difficult). The range of allowed STOP times is 300–700 ms after trial onset. Thus, by design, the expected overall success rate for each individual is approximately 50%. The primary indicator of better response inhibition efficiency is thus not the STOP success rate but instead is a shorter SSRT, calculated as the difference between the average GO (finger lift) time during GO trials and the STOP cue time during STOP trials.

#### Race-car version

We previously customized the Slater-Hammel into a child-friendly race-car version, executed using Presentation (v. 23.1; Neurobehavioral Systems).^29^ Participants faced the monitor display while seated in a comfortable chair with the ulnar aspects of both arms and hands resting on a body-surrounding pillow. The fully extended index finger of the dominant hand operated a game controller by pressing down to move the car on the track toward a finish line at 800 ms. The GO action required participants to use the first dorsal interosseous (FDI) to lift the finger when the race car was as close to 800 ms as possible.

In our prior study, participants practiced GO-only, STOP-only, and mixed GO/STOP trials. Then GO times were evaluated during 80 trials using TMS (see below).^17^

In the present study, participants practiced GO-only, then performed a simple reaction time block with 40 trials. Participants then practiced STOP-only and mixed GO/STOP trials, then performed the response inhibition task during 96 trials with a 3:1 GO:STOP ratio.

**Figure 1.**
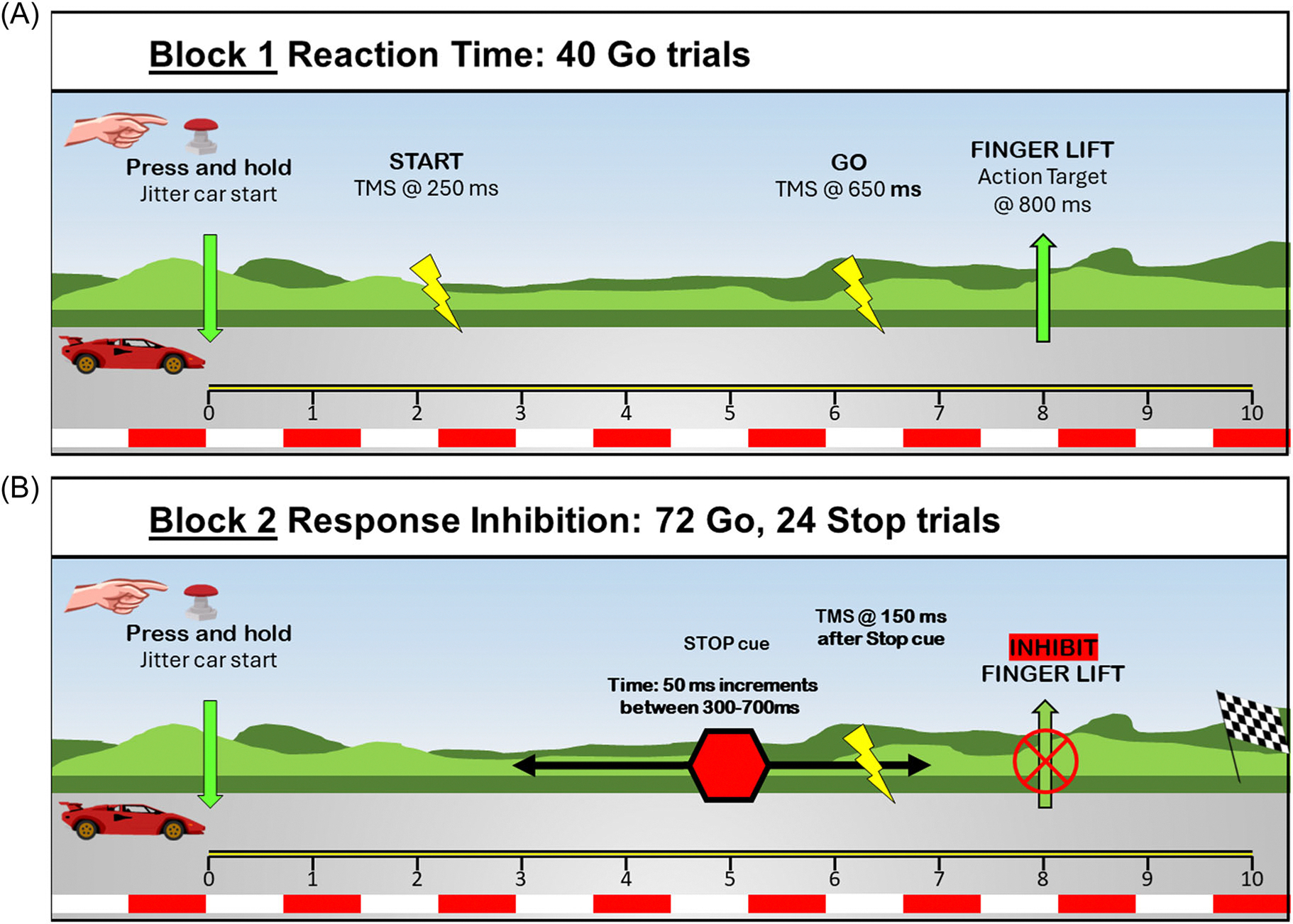
Race-car Slater-Hammel task. (A) Simple reaction time task, 40 GO-only trials after 12 practice trials, in which participants press the game controller button with their right index finger and hold to drive the car, then activate the first dorsal interosseous (FDI) to lift the finger as close to 800 ms as possible. Trial-by-trial feedback was as follows: 700 to 800 ms—“Good Job!”; <700 ms—“Too Early!”; >800 ms—“Too Late!” TRUM: TMS over left motor cortex, right FDI motor evoked potential. Randomized pulses, at resting motor threshold intensity, either at 250 ms, “START,” or 650 ms, “GO.” (B) Response inhibition task. After 8 STOP-only and 12 mixed GO/STOP practice trials, participants engaged in a response inhibition task. The task was the same as the simple reaction time, consisting of 72 trials (36 START, 36 GO) but with 24 STOP trials randomly intermixed. STOP cue: dynamic based on success/failure; the car stops between 300 and 700 ms (see Methods section). Successful response inhibition is achieved by withholding finger lift at 800 ms and continuing to press down until >1000 ms, when the checkered flag appears. Trial-by-trial STOP feedback was as follows: <1000 ms—“Too Early!”; >1000 ms—“Good Job!” GO to STOP is a 3:1 ratio. See text for descriptions of stop-signal reaction time.

The GO-only and response inhibition blocks are shown in Figure 1.

### TMS Procedures

Both sites used Magstim 200 TMS (Magstim) systems with a Bistim module and a round 90-mm coil, as well as identical amplifiers and filter settings.^17^ Surface electromyography (EMG) electrodes were placed on the FDI, the neighboring metacarpophalangeal joint, and a ground site on the forearm. EMG data were recorded using Signal software (Signal V6). Data extraction and processing were blinded to diagnosis.

Resting motor threshold (RMT) was determined as described in our prior study, with the TMS coil placed flat at the vertex, the handle directly posterior, and the current counterclockwise. The operator administered single TMS pulses starting at 20% maximal stimulator output, increasing by 10% until a consistent motor evoked potential (MEP) was observed from surface EMG recording in the right FDI muscle, then decreasing the intensity by increments of 1%–2% until tracings were flat or MEPs were less than 50 μV. At this point the operator administered additional TMS pulses, adjusting the intensity until three pulses produced no MEP and three produced an MEP of approximately 50 μV.^11^

### TRUM

#### Simple reaction time block, 40 trials

TRUM, that is, the amplitude of the MEP from the FDI activated during the tasks, was assessed by stimulating M1 with the TMS coil at three informative times. In our prior study, the operator administered single-pulse TMS at 20% above the participant’s RMT. GO TRUM was evoked at 650 ms, that is, 150 ms prior to the anticipated GO. There was no simple reaction time block—only response inhibition (see below).^17^ In the present study, the operator administered single-pulse TMS at RMT for greater comfort of participating children. In addition to the GO pulses at 650 ms (20 trials), to capture task engagement prior to the critical GO action, we added 20 additional trials to measure TRUM at 250 ms, designating this time point “START.” TMS pulses (one per trial) START versus GO were randomized. Any trial with early (pre-TMS pulse) actions were replaced.

#### Response inhibition task

As in the prior study, STOP TRUM was evoked 150 ms after the STOP cue.^17^ The operator again administered single-pulse TMS at RMT. Trial pulse times were randomized, with 72 GO (lift finger) and 24 STOP (inhibit finger lift). TMS pulses were administered at 250 ms (START; 36 trials), 650 ms (GO; 36 trials), or 150 ms after STOP cue (STOP; 24 trials), in random order.

#### Additional modifications

To maintain attention and make the task slightly more challenging, we introduced a jitter timing feature at the onset of movement of the race car. Because TMS pulses always occur 150 ms after STOP cues, the participant could realize that an early TMS pulse (250 ms) would never be a STOP trial. To eliminate this possibility, we interspersed at random four additional “decoy STOP” trials with a TMS pulse at 250 ms prior to a STOP cue. We excluded these four decoy trials from analysis.

### Statistical Analysis

#### Univariate analyses of behavior, motor function, motor physiology, and clinical/demographic variables

Motor, physiologic, behavioral, SSRT, and demographic data were compared across diagnostic groups using Welch’s *t*-tests, *χ*^2^, and Fisher’s exact test as appropriate.

#### Performance by diagnosis: Repeated measures regression analyses of times of going and stopping

For GO-only, simple response time trials, we compared diagnostic group performance based on the timing of the GO (finger lift) across all trials (excluding the practice trials). We used mixed model, repeated measures regression with the GO time as the dependent variable. In addition, we compared the proportion of successful trials (finger lift between 700 and 800 ms) using logistic regression.

In the response inhibition trials, we performed the same regression analyses for GO (finger lift) times, STOP cue (car stop) times, and proportions of successful STOP trials.

In all regressions, in addition to the variable diagnosis (ADHD, TD), we included sex (male, female), age, and city (Cincinnati, Baltimore). Reported lift times for diagnoses are least squares means (LS means) estimates, with standard errors (SE).

SSRT is the mean finger lift time during GO trials minus the mean cue time during STOP trials. We calculated this SSRT for each individual, then compared groups using a Welch’s *t*-test.

#### Response inhibition by diagnosis: Repeated measures analyses of TRUM

The primary outcome of interest for this study was TRUM during the response inhibition task. Because stimuli occur at the participant’s RMT, the expected rest MEP amplitude average would be 0 mV. However, since the participant is preactivating M1 in preparation to use the target muscle for the task, the expected action-related MEP amplitude for each trial is >0 mV and reflects motor cortex activity at that time point. We quantified TRUM from TMS pulses administered at two time points in block 1 (simple reaction time—GO-only): (1) START and (2) GO. We quantified TRUM at three time points in block 2 (response inhibition—GO/STOP): (1) START, (2) GO, and (3) STOP.

The primary planned analysis for TRUM was performed on all trials using repeated measures mixed models with participant as a random effect and log-transformed MEP amplitude as the dependent variable.^17^ This method accounts for nonnormality and intrasubject variability of MEPs.^17,35,36^ The primary experimental variables were diagnosis (ADHD vs. TD) and task (START, GO, STOP). We hypothesized that TRUM would differ by task difficulty, with STOP > GO > START, and that the task effect on TRUM would depend on diagnosis (interaction term). All STOP trials were included to capture effects related to the STOP cue.

As for the reaction time analyses, we included age, sex, and city. We also included pre-TMS background EMG (captured during a 50-ms epoch prior to each TMS pulse) in all models. All models were analyzed using SAS statistical software version 9.4 (SAS Institute Inc.). See Supporting Information files for model statements.

After the primary analyses, we conducted a prespecified post hoc comparison across ADHD versus TD for START, GO, and STOP to test whether diagnosis effects were robust within trial types. We hypothesized that the largest difference would be for STOP.

#### Response inhibition STOP-TRUM, dimensional analysis of ADHD rating scales and SSRT

We performed a dimensional analysis, replacing the categorical ADHD versus TD diagnoses with parent-rated ADHD severity scales and with SSRT as continuous variables. We hypothesized the STOP-TRUM would be lower among children with higher ADHD scale scores and longer SSRTs.

#### Exploratory analyses of TRUM and diagnosis (see Supporting Information file)

##### Movement timing and TRUM

MEP amplitudes would be expected to be larger when a finger lift occurred closer to the TMS pulse.^23^ Therefore, for each GO trial, we calculated a “move time” (GO time minus TMS pulse time) and modeled TRUM as a function of diagnosis and move time.

##### TRUM for GO versus successful-STOP-only

Limiting the analysis to successful STOP reduces statistical power, a pitfall since only 25% of all trials require stopping. We conducted an exploratory subanalysis of TRUM, eliminating START trials, restricting STOP to successful STOPs only, and modeling TRUM as a function of diagnosis and trial type.

##### TRUM for GO versus failed-STOP-only

To determine whether a failed STOP (in which the GO is not inhibited) differs from a GO, we conducted an exploratory subanalysis of TRUM, eliminating START trials, restricting STOP to failed STOPs only, and modeling TRUM as a function of diagnosis and trial type.

##### TRUM for GO (simple reaction vs. response inhibition)

We expected that GO-TRUM might reflect the expectation of stopping, and thereby GO-TRUM might differ in block 1, simple reaction, versus block 2, response inhibition. We conducted an exploratory subanalysis of TRUM, including GO trials across both blocks, eliminated STOP trials, and modeled TRUM as a function of diagnosis and “GO-type.”

##### Dimensional analysis

We expected that diagnostic severity and response inhibition efficiency might be reflected differently in START, GO, and STOP trials. We therefore re-analyzed the response inhibition trials, stratifying by the three trial types. We modeled TRUM as a function of ADHD scores and SSRT.

## Results

### Participants

The cohort included 79 8-to-12-year-old, right-handed children (52% with ADHD; 47% female). Demographic and clinical variables were compared by group (Table 1).

### Performance—Reaction Times

#### Simple reaction time (GO-only): performance does not differ in children with ADHD

Children with ADHD and TD control peers performed similarly during the GO-only trials (where no stopping was required).

GO times did not differ between ADHD and TD. Age, city, and gender were also nonsignificant (all *p* > 0.10). The GO success rate, that is, the proportion of GO actions (finger lifts) within the target interval of 700–800 ms, did not differ by diagnosis.

##### Additional findings

GO times occurred earlier in START trials (TMS pulses at 250 ms) (mean 775.7 ms, SE 3.7 ms) versus GO trials (TMS pulses at 650 ms) (mean 803.7 ms, SE 3.7 ms) (regression test statistic for diagnosis F1,3003.333 = 87.1; *p* < 0.001). However, we found no evidence that this depended on diagnosis (interaction *p* = 0.16). Age, city, and gender were also nonsignificant (all *p* > 0.10). GO times remained consistent over the course of the task (trial number effect *p* = 0.21).

#### Response inhibition (GO/STOP): Performance is worse in children with ADHD

SSRTs were significantly longer in children with ADHD. This indicates worse performance in children with ADHD (Table 2).

In children with ADHD, finger lift occurred later, and mean car stop (STOP cue) times occurred earlier, although neither alone reached significance. For both groups, mean

GO times and variability were greater in the response inhibition segment than in the preceding GO-only segment (Table 2).

##### Additional findings

GO times occurred earlier for TMS pulses at 250 ms (mean 801 ms, SE 5.1 ms) versus TMS pulses at 650 ms (mean 838.2 ms, SE 5.2 ms) (F1,5428.173 = 160.432; *p* < 0.001). However, this did not differ by diagnosis. Finger lift times during GO trials shortened (improved) as the game progressed (F1,5476.337 = 26.651; *p* < 0.001), but this did not differ between diagnoses (*p* = 0.902). This finding validated that motivation and effort levels remained high in participants in both groups. Both groups’ success rates of approximately 60% are acceptable (sufficiently close to 50%). Of note, this rate of STOP success combined with the slightly longer GO times (and less GO success) in the response inhibition task may also indicate some tendency to wait for the STOP cue.

### Task-Related Up-Modulation in Motor Cortex (M1 TRUM)

#### Simple reaction time (GO-only): TRUM does not differ in ADHD

**Figure 2.**
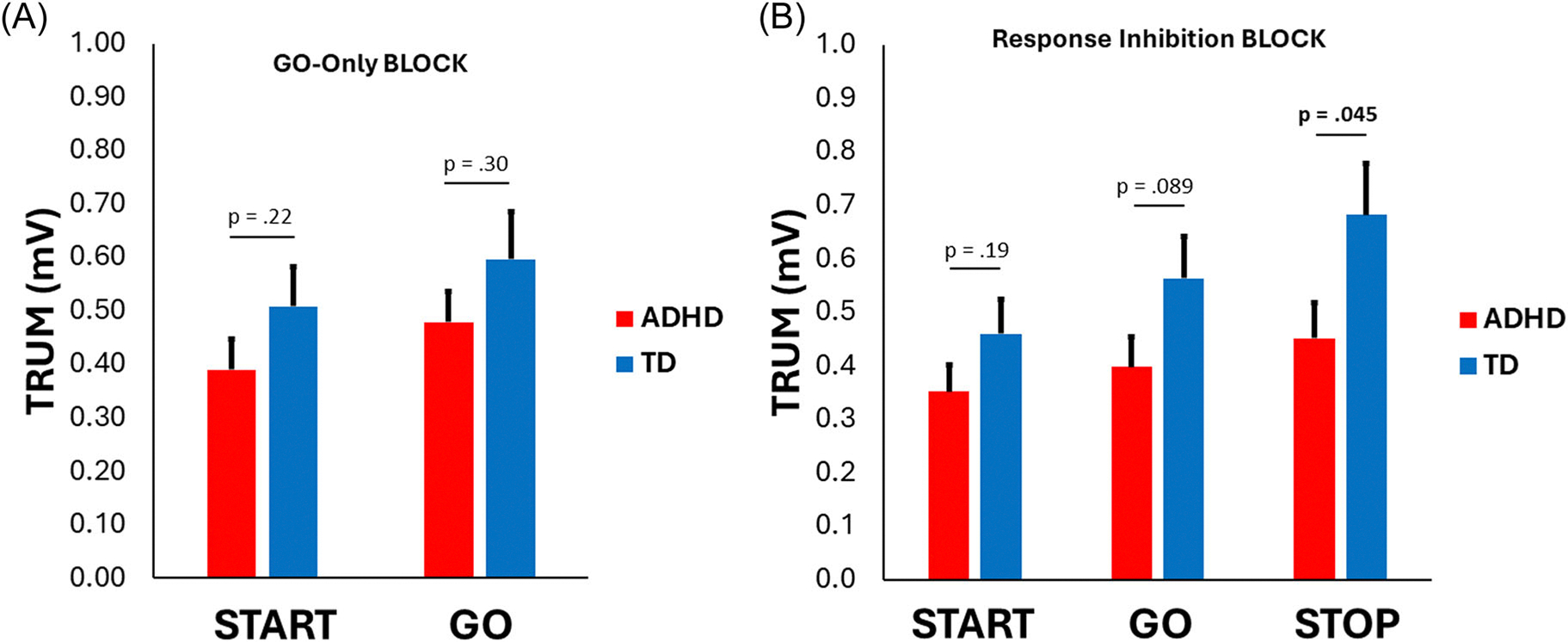
TRUM is task-related up-modulation of M1 motor evoked potential amplitudes, in mV, from surface electromyography (EMG) in hand (see methods). TMS pulses were administered as shown in Figure 1. (A) Simple reaction task: TRUM GO > START (*p* < 0.001). The effect of task does not depend on diagnosis. (B) Response inhibition task: TRUM STOP > GO > START (*p* < 0.001 for each). The effect of task depends on diagnosis (*p* < 0.001). Post hoc comparisons as shown. ADHD, attention-deficit/hyperactivity disorder; mV, millivolts peak-to-peak activity on EMG tracing; TD, typically developing control children; TRUM, task-related up-modulation of M1 excitability.

In the simple reaction time task (GO-only), as expected, TRUM was significantly greater (larger MEP amplitudes) at 650 ms (GO) versus 250 ms (START). This effect of trial type did not differ by diagnosis. Diagnosis alone did not reach significance. Post hoc comparisons within START and GO trials showed no difference by diagnosis (Table 2 and Figure 2A).

#### Response inhibition (GO/STOP): STOP-TRUM is diminished in ADHD

In the response inhibition task (GO/STOP), the times of all individual trials’ finger lift responses varied by task (trial type) (F2,7267 = 127.064; *p* < 0.001). Failed STOP trials clustered around the target GO time of 800 ms.

TRUM differed depending on trial type: STOP > GO > START. That is, the more difficult the task was, the greater the TRUM. This differential task effect depended on diagnosis (F2,7267 = 6.34; *p* = 0.002) and was significantly less prominent in ADHD. Diagnosis alone approached statistical significance (F1,73 = 2.923; *p* = 0.09). Post hoc pairwise comparisons showed TRUM differences in ADHD were greatest during STOP trials (Table 2 and Figure 2B).

#### STOP-TRUM diminishes at higher ADHD symptom severity

**Figure 3.**
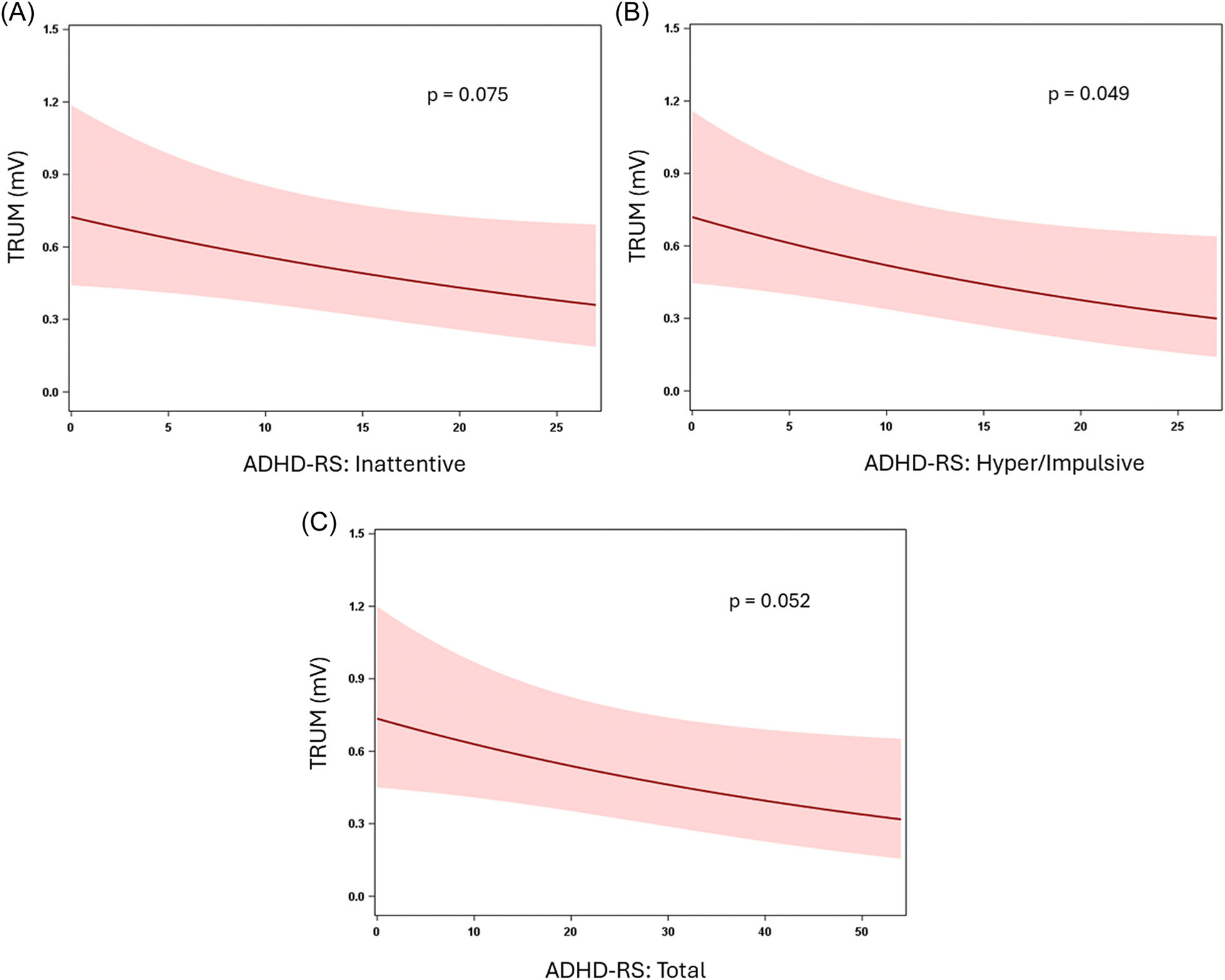
Task-related up-modulation (TRUM) is the vertical distance from zero to the solid line. Worse attention-deficit/hyperactivity disorder (ADHD) severity, as assessed by the ADHD rating scale (ADHD-RS), is associated with less TRUM during STOP trials in the response inhibition task. Shaded regions indicate standard errors. (A) ADHD-RS inattentive subscale. (B) ADHD-RS hyperactive/impulsive subscale. (C) ADHD-RS total combined.

STOP-TRUM, which was significantly diminished in ADHD, was re-evaluated after removing the categorical diagnoses and substitutign the ADHD rating scale (total, subscales). STOP-TRUM was significantly lower among children with higher ADHD-RS hyper/impulsive subscale scores (F1,64.910 = 4.018, *p* = 0.049) and approached significance for the inattentive subscale (F1,64.92 = 3.29, *p* = 0.075) and total ADHD-RS (F1,64.92 = 3.90, *p* = 0.052) (Figure 3). There was no significant association with SSRT (F1,72.9908 = 0.264, *p* = 0.61).

### Exploratory Analyses

#### Movement timing and TRUM

During the simple reaction time task, in TD children, MEP amplitudes were larger, as expected, in trials in which GO (the finger lift) occurred closer to the time of the TMS pulse (650 ms). This effect may be less robust in ADHD (Supporting Information S1: Figure 1).

#### TRUM for GO versus successful-STOP-only

The influence of task (TRUM STOP > GO; *p* < 0.001) was similar in this subanalysis (Supporting Information S1: Figure 2A). The influence of diagnosis (TRUM ADHD < TD) approached significance (*p* = 0.08).

#### TRUM for GO versus failed-STOP-only

The influence of task (TRUM STOP > GO; *p* < 0.001) was similar in this subanalysis (Supporting Information S1: Figure 2B). The effect of task depended on diagnosis (*p* = 0.004). Influence of diagnosis alone (TRUM ADHD < TD) was also significant (*p* = 0.025).

#### TRUM for GO (simple reaction vs. response inhibition)

The influence of diagnosis was not significant (Supporting Information S1: Figure 3).

#### Dimensional analysis

The influence of task (TRUM STOP > GO > START) was significant (*p* < 0.001). However, this influence diminished among children with higher ADHD scores and among children with longer (worse) SSRT (all interaction terms *p* < 0.001) (Supporting Information S1: Figure 4).

## Discussion

Our findings show that, for 8-to-12-year-old children, selecting-to-stop (STOP) activates M1 more than participation (START) or preparing-to-go (GO) activates M1. This difference (TRUM STOP > GO > START) was present in both children with ADHD and their TD peers, but the differences were larger among TD children. Within tasks, the largest difference in TRUM between ADHD and TD children occurred specifically during STOP trials. This difference, while modest, was corroborated by a dimensional analysis in which STOP-TRUM was reduced more among children with greater ADHD symptom scores. If validated, this suggests that TRUM, particularly during response inhibition, may serve as a useful biomarker of behavioral diagnoses or diagnostic subgroups in which individual children have impaired response inhibition.

In this study, we extended and clarified prior findings of reduced response inhibition–associated TRUM in ADHD,^17^ showing a complex relationship between the ADHD diagnosis, response inhibition performance, and TRUM. First, we showed that across all children, actions (task engagement—START; action preparation—GO; cued stopping—STOP) induce significant TRUM. Second, we showed that the largest TRUM was observed during STOP, corresponding to greater task difficulty. Third, we found that, specifically under the cognitive load of response inhibition (and not simple reaction time), children with ADHD showed both worse performance (longer SSRTs) and less TRUM than did TD controls. To understand this finding, it is important to note that in this task, only the SSRT, which was longer in children with ADHD, is the indicator of response inhibition insufficiency. The comparable STOP success rates across groups occurs by design—the dynamic success-based timing of the STOP cue generates an approximately 50% success/fail rate in STOP trials (Table 2). Finally, we showed that reduced TRUM occurring during STOP trials was associated with greater parent-rated ADHD symptom severity.

This study supports ongoing efforts to develop and measure quantitative biomarkers of ADHD. The heterogeneity of ADHD symptoms, impaired function, and biology predicts that individual putative biomarkers are unlikely to fully differentiate children with ADHD from their TD peers. In addition, the comorbidity profiles and treatment responses vary widely among children with ADHD. Future studies could recruit these groups and characterize response inhibition TRUM, or other TRUM,^37^ possibly yielding biomarkers that account for differences in short-term therapeutic responses and long-term outcomes in various ADHD subgroups.

Our analysis of STOP-TRUM versus symptom severity used ADHD rating scale scores in addition to the categorical diagnosis. Importantly, the TRUM/severity relationship was in the expected direction—less STOP-TRUM among children with higher, more severe rating scale scores. An association with STOP-TRUM did not extend to SSRT performance. However, in the full model, the robust effect STOP > GO > START was greater (see supplemental file) among children with efficient response inhibition (shorter SSRTs). Further exploration of these complex interactions across and within diagnoses is warranted.

In summary, TD children appear to activate M1 as soon as either a reaction time or a response inhibition task begins but before a response is required. When approaching an anticipated response, they further up-modulate M1 excitability. In the response inhibition task, the introduction of a STOP cue prior to the anticipated GO response activates their M1 to an even greater extent. In ADHD, these effects are less robust and less differentiated.

### Limitations

In studies of this nature, convenience samples meeting strict inclusion criteria may not represent the broad spectrum of children with clinically rated neurobehavioral diagnoses. We excluded children with commonly co-occurring diagnoses such as anxiety, depression, or autism spectrum disorder, so our findings may not generalize to those groups. However, the lack of any site effects in children recruited in two geographically distinct cities supports some generalizability of our findings for “uncomplicated” ADHD. In our experiments, the overall number of response inhibition trials was relatively small. In designing tasks for inattentive young children, the greater precision with more trials has the trade-off of inducing boredom, possibly confounding findings. Notably, we found no evidence that performance declined toward the end of the experimental blocks. Finally, we cannot exclude that order of blocks influenced our findings. The GO-only block was always performed first, in hopes of more extensively priming the expectation to GO. Also, we surmised that it was more accurate to quantify GO-only TRUM in children who had not yet been exposed to the instruction of possibly having to stop. Finally, we chose to use the more diffuse round coil and not the more focal Figure-8 coil for practical reasons—young children move around more than adults. Thus, although we report TRUM MEPs from the muscle engaged in the response inhibition task, this readout may reflect activity outside of M1 to a greater degree than the Figure-8 coil.

## Conclusions

TMS has shown promise as a minimal-risk experimental technique in multiple prior studies of neurobehavioral diagnoses in children.^34^ In this study, we found that TMS-evoked TRUM is highly sensitive to task, with STOP > GO > START TRUM. Additionally, we found that this task-based variation in TRUM was reduced in children with ADHD, so that children with ADHD showed particularly reduced TRUM during stop trials, i.e., those requiring inhibition of a prepotent motor response. In contrast, there was no significant effect of ADHD diagnosis on TRUM during performance of a simple reaction time (“GO-only”) task. Further, we found that elevated parent ratings of ADHD symptom severity were associated with less TRUM during STOP trials. Complementing most prior TMS “rest-M1” studies, this study supports ongoing efforts to incorporate “active-M1” for tasks in which function tends to be impaired. Using this approach, our findings provide evidence that TRUM during response inhibition may be considered as a quantitative, brain-based marker of impaired cognitive control in children with ADHD.

## Supplementary Material

1

Supporting Information

Additional supporting information may be found online in the Supporting Information section at the end of the article.

## Figures and Tables

**Table 1.** Demographics and clinical ratings.

Characteristic	ADHD *n* = 41	TD control *n* = 38	*p* value

	*n (%)*	*n (%)*	
Gender			
Female	17 (42%)	20 (53%)	0.32
Male	24 (59%)	18 (47%)	
Race			
Asian	2 (4.9%)	4 (11%)	0.83
Black or African American	5 (12%)	4 (11%)	
More than one race	3 (7.3%)	2 (5.3%)	
White	31 (76%)	28 (74%)	
Ethnicity			
Hispanic or Latino	3 (7.3%)	0 (0%)	0.09
Not Hispanic or Latino	38 (93%)	38 (100%)	
	*Mean* (*SD*)*S*	*Mean* (*SD*)	
Age	10.3 (1.4)	9.8 (1.2)	0.09
Clinical ratings			
ADHD-RS inattentive	17 (5)	3 (3)	**<0.001**
ADHD-RS hyper/impulsive	13 (6)	2 (3)	**<0.001**
ADHD-RS total	30 (10)	5 (6)	**<0.001**

*Note*: Group comparison *p* values from Welch’s f-tests, *χ*^2^, and Fisher’s exact test proportions as appropriate. Bold font indicates statistical significance in regression model.

Abbreviations: ADHD, attention-deficit/hyperactivity disorder; ADHD-RS, ADHD rating scale; SD, standard deviation; TD, typically developing.

**Table 2.** Response inhibition: Regression results for performance and TRUM, by diagnosis.

Characteristic	ADHD, *n* = 41	TD control, *n* = 38	*p* value

*Performance*			
Simple reaction time (GO-only)			
GO success, %	51.1%	47.5%	0.36
GO time (finger lift) in ms, LS mean (SE)	789 (4.9)	790 (4.8)	0.90
Response inhibition (GO/STOP)			
Stop signal reaction time in ms, mean (SD)	313 (66)	284 (48)	**0.029**
GO success %	36.4%	42.6%	**0.044**
STOP success %	62.0%	61.7%	0.91
GO time in ms, LS mean (SE)	827 (7.1)	814 (6.9)	0.16
STOP cue time in ms, LS mean (SE)	508 (9.7)	528 (9.4)	0.15
*TRUM*			
Simple reaction time (GO-only)			
*TRUM: post hoc within task*			
START (250 ms) mV LS mean (SE)	0.39 (0.058)	0.51 (0.075)	0.22
GO (650 ms) mV LS mean (SE)	0.48 (0.071)	0.60 (0.088)	0.30
Response inhibition (GO/STOP)			
*TRUM: post hoc within task*			
START (250 ms) mV LS mean (SE)	0.35 (0.050)	0.46 (0.064)	0.19
GO (650 ms) mV LS mean (SE)	0.40 (0.057)	0.56 (0.079)	0.089
STOP mV LS mean (SE)	0.45 (0.065)	0.68 (0.096)	**0.045**

*Note*: STOP success percentage does not indicate response inhibition efficiency or task difficulty (see text).

Abbreviations: ADHD, attention-deficit hyperactivity disorder; LS mean, least squares mean, repeated-measures mixed-model regression estimates; mV, millivolt; SD, standard deviation; SE, standard error; SSRT, stop-signal reaction time; TD, typically developing; TRUM, task-related up-modulation. Bold font indicates statistical significance in regression model.

## Data Availability

Per policy of the National Institute of Health, all data will be available after publication through the NIMH Data Archive (https://nda.nih.gov).
