# Supplementary material for: In children with attention-deficit/hyperactivity disorder, less task-related up-modulation of motor cortex during response inhibition
Source: Ann Child Neurol Soc. Author manuscript; Available in PMC 2026 Jul 29. (PMC13359149; doi:10.1002/cns3.20101)
Supplement: 1 [file NIHMS2189082-supplement-1.docx]

**Supplemental file**

**Models for Performance (reaction time, finger lift time**)

See methods for details. Mixed Models, repeated measures regression also include age (continuous), sex (male, female), and site (Cincinnati, Baltimore). The following table lists the questions and the regression model variables.

| **Question** | **Model** |
| --- | --- |
| **PERFORMANCE – Reaction Time (Go-only)** | Go-only trials: finger lift between as close to 800 ms as possible |
| Is reaction time (finger lift time) different in ADHD? | RT = Dx (ADHD, TD) |
| Is reaction time different based on TMS pulse time at 250 (Engage) vs. 650 (preparing to go)? | RT = PulseTime |
| Does PulseTime difference depend on diagnosis? (interaction) | RT = PulseTime \| Dx |
| Is there a learning (trial number) effect on RT? | RT = TrialNumber |
| *Success*  *Go trials: finger lift between as close to 800 ms as possible* |  |
| Does the probability of success differ in ADHD? | P(Success) = Diagnosis – logistic |
| **PERFORMANCE – Response Inhibition (START, GO, STOP)** |  |
| Is Go-time (Go trial finger lift time) different in ADHD? | RT = Dx (ADHD, TD) |
| Is there a learning (trial number) effect on finger lift time in Go trials? | RT = TrialNumber |
| *SUCCESS*  *Go trials: finger lift between as close to 800 ms as possible*  *Stop trials: withhold finger lift until after 1000 ms (checkered flag)* |  |
| Does the probability of successful Go trials differ in ADHD? (Finger Lift between 700 and 800 ms) | P(GO Success) = Diagnosis – logistic |
| Does the probability of successful STOP trials differ in ADHD? (Finger lift after 1000 ms) | P(STOP Success) = Diagnosis – logistic |

**Models for TRUM (MEP amplitude)**

See methods for details. Mixed models repeated measures regressions also include age (continuous), sex (male, female), site (Cincinnati, Baltimore), and pre-TMS-pulse artifact. The following table lists the questions and the regression model variables.

| **Question** | **Model** |
| --- | --- |
| **TRUM - – Reaction Time (Go-only)** | TRUM = task-related up-modulation, motor evoked potential amplitude evoked at RMT |
| *pre-movement artifact is displacement in EMG tracing during 50 ms epoch prior to TMS pulse* |  |
| Is pre-movement artifact different in ADHD? | Artifact = Diagnosis |
| Preparing to move activates motor cortex |  |
| Does pre-movement motor cortex activation depend on timing of pulse versus time of finger lift? (for movetime subtract finger lift from TMS time for each trial) | MEP = Movetime |
| Does the Movetime effect on MEPs depend on diagnosis? | MEP = Movetime \| Dx |
| Is TRUM (MEP) different based on TMS pulse time at 250 (START) vs. 650 (preparing to GO)? | MEP = TrialType |
| Does the effect of START versus GO depend on diagnosis? | MEP = TrialType \| Diagnosis |
| **TRUM - – Response Inhibition**  **(START, GO, STOP)** |  |
| Is pre-movement artifact different in ADHD? | Artifact = Diagnosis |
| Is TRUM (MEP) different based on TMS pulse time at 250 (START) vs. 650 (preparing to GO) vs STOP (150 ms after cue)? | MEP = TrialType |
| Does the effect of START versus GO versus STOP on TRUM depend on diagnosis? | MEP = TrialType \| Diagnosis |
| Planned post-hoc within-trial-type, across diagnosis | MEP_STOP = Diagnosis  MEP_GO=Diagnosis  MEP_START=Diagnosis |
| Dimensional Analyses | MEP_STOP = ADHD_rating_scale  MEP_STOP = SSRT |
|  |  |
| Additional TRUM analyses (filtered by trial subtypes) |  |
| Does the effect of GO versus Successful STOP on TRUM depend on diagnosis? | MEP = TrialType \| Diagnosis |
| Does the effect of GO versus Failed STOP on TRUM depend on diagnosis? | MEP = TrialType \| Diagnosis |
| Does effect of GO-Only GO vs GO/STOP-GO on TRUM depend on diagnosis? | MEP = TrialType \| Diagnosis |

**Additional analyses and figures**

1. **Movement Timing and TRUM**

**Supplemental Figure 1. TRUM prior to movement**


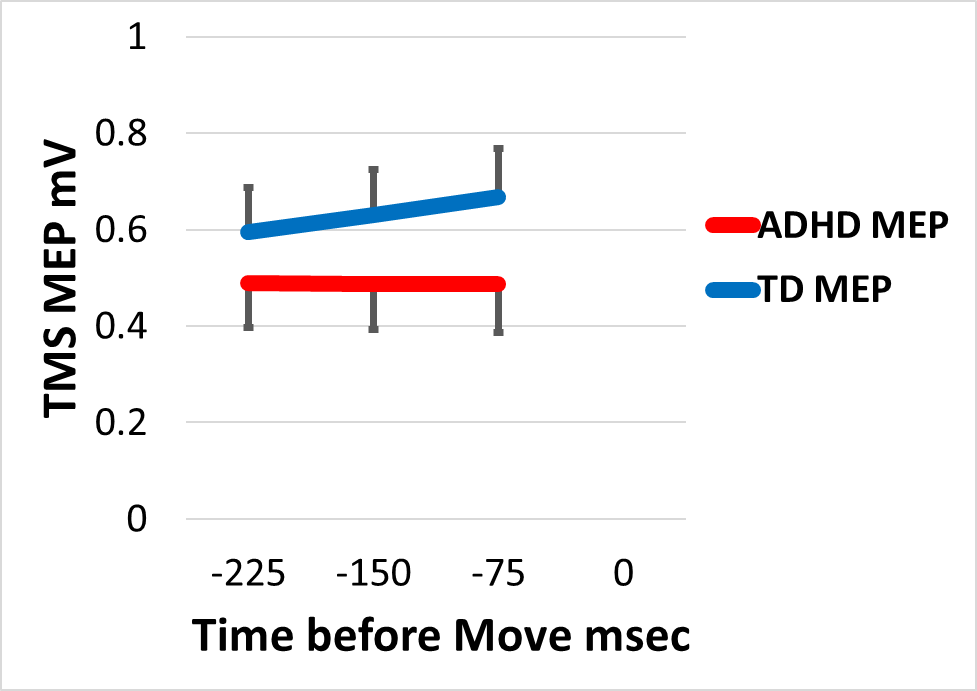


**Supplemental Figure 1. Effect of movement preparation on TRUM in Response inhibition**

The X axis is the time difference between the TMS pulse in the GO trials (650 ms) and the finger lift, in each trial, in the Block 1 Reaction Time Task. The Y axis is TRUM. The mixed model regression is performed as described in the methods. Lines show regression LS Means estimates and standard errors for children with attention deficit hyperactivity disorder (ADHD) and typically developing (TD) controls. The expected finding of larger MEP amplitudes when TMS pulses occur closer to the finger lift (GO movements), shown in healthy adults (Chen *et al.*, 1998), was not significant in children overall (MEP = Movetime; p = 0.16; not shown). However, including diagnosis and interaction (MEP = Movetime|Dx; see regression output below) showed MEP reflected movetime, diagnosis at the trend level, and a trend-level interaction. Repeating the same analysis Response Inhibition Task showed no significant effect of time (p = 0.18).

***Regression - Solution***

1. **TRUM for GO versus Successful-STOP-only – 2A**

**Supplemental Figure 2A. TRUM during preparing-to-go versus successful-stop**


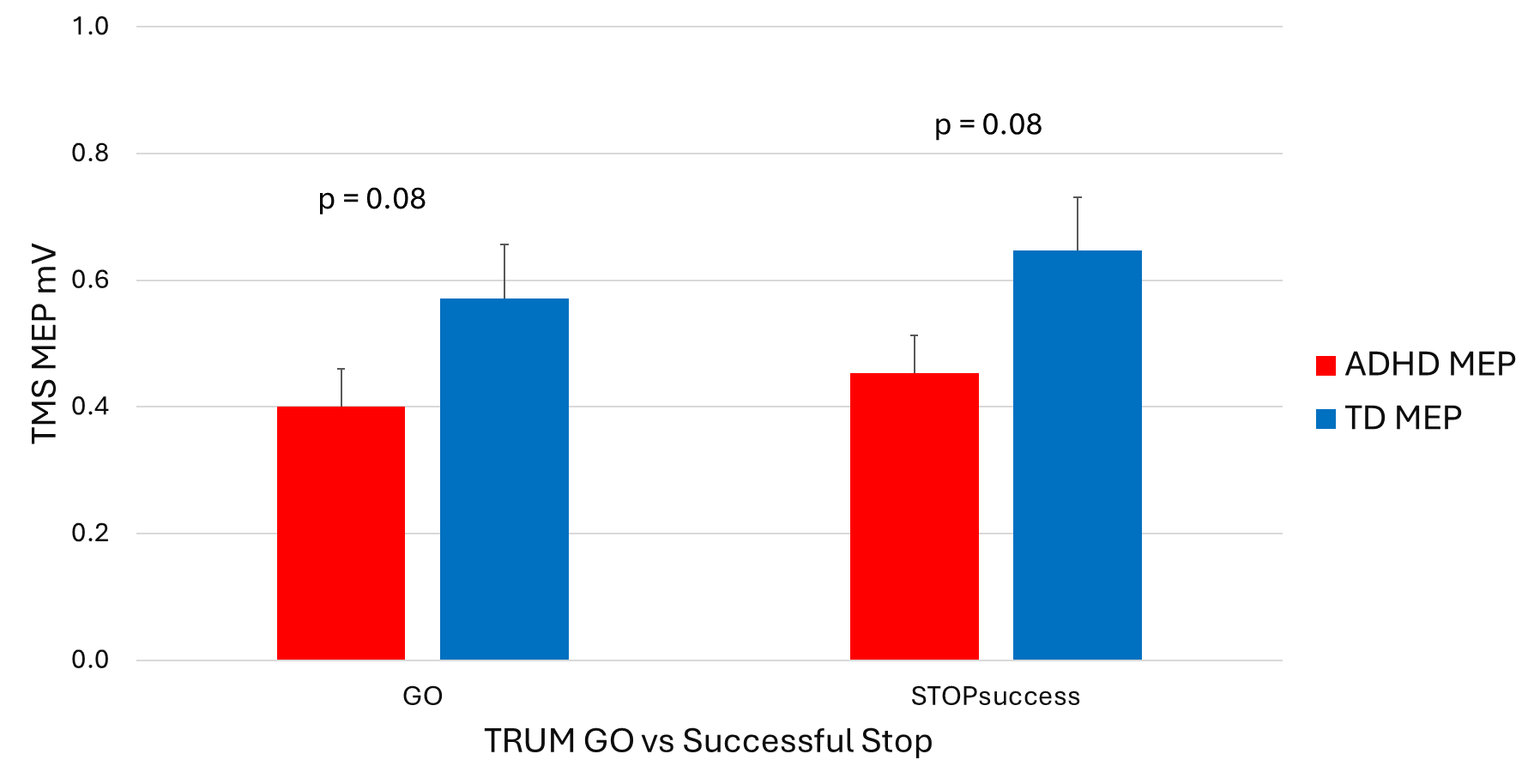


**Supplemental Figure 2A. TRUM restricted to GO vs. successful STOP**

In this model, START trials are removed, so there is no accounting for effects of game participation separate from selecting Go or Stop. Failed stops are also removed, so that there is no finger lift until after 1000 ms. Response inhibition TRUM from Experimental Block 2 was re-estimated comparing GO (TMS at 650 ms) to Successful Stop only (i.e., removing failed STOP).

***Regression solution***

***Type III***

***Post hoc***

**2B TRUM for GO versus failed stop**

**Supplemental Figure 2B. TRUM during preparing-to-go versus failed-stop**

**
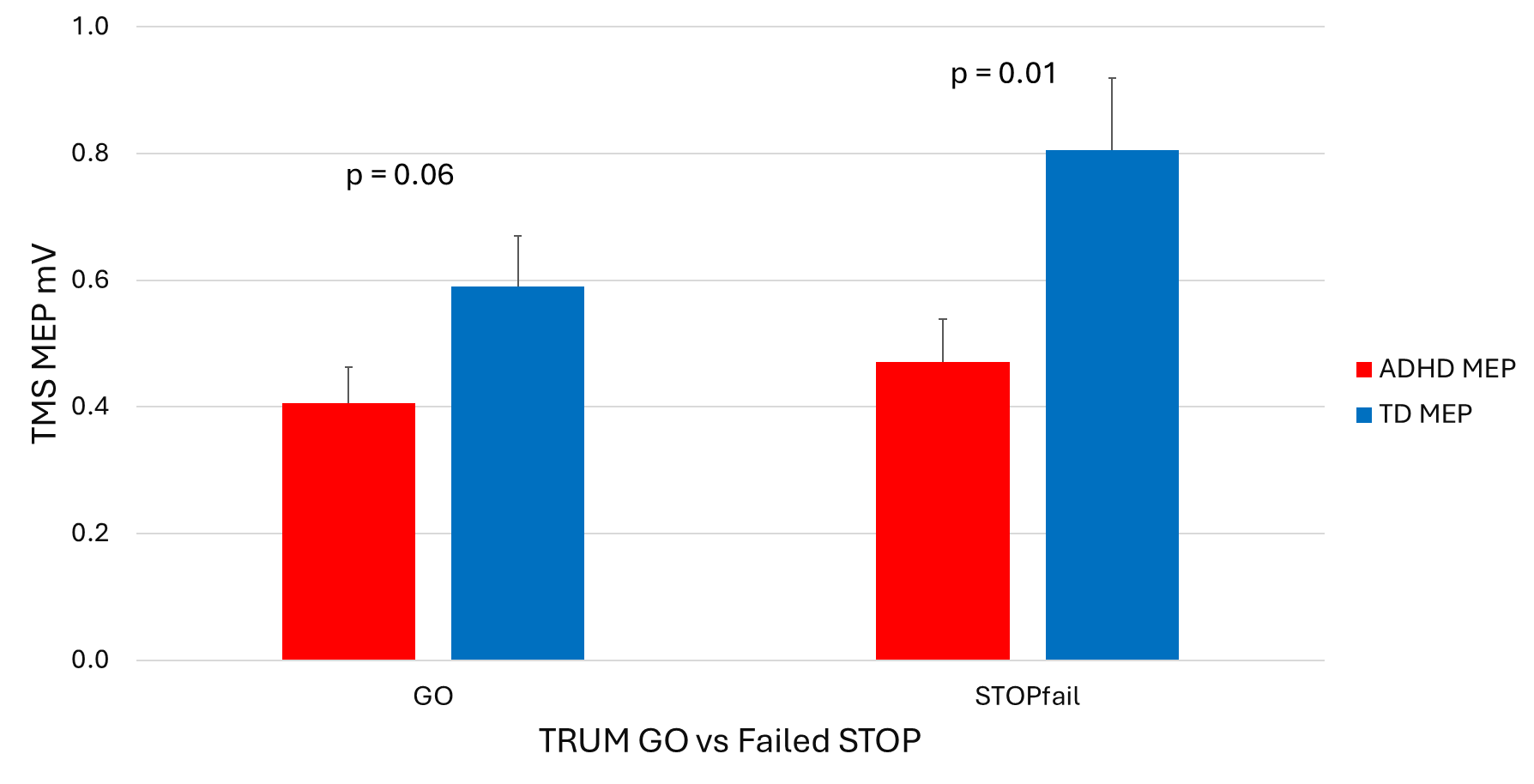
**

**Supplemental Figure 2B. TRUM restricted to GO vs. failed STOP**

In this model, START trials are removed, so there is no accounting for effects of game participation separate from selecting Go or Stop. Successful stops are also removed, so that the finger lifts occurred inappropriately before 1000 ms. Response inhibition TRUM from Experimental Block 2 was re-estimated comparing GO (TMS at 650 ms) to Failed Stop only (i.e., removing successful STOP).

***Solution***

***Type III***

***Post hoc***

1. **TRUM for GO during simple reaction time vs. GO during response inhibition task**

**Supplemental Figure 3. GO-TRUM during simple reaction time versus during response inhibition**


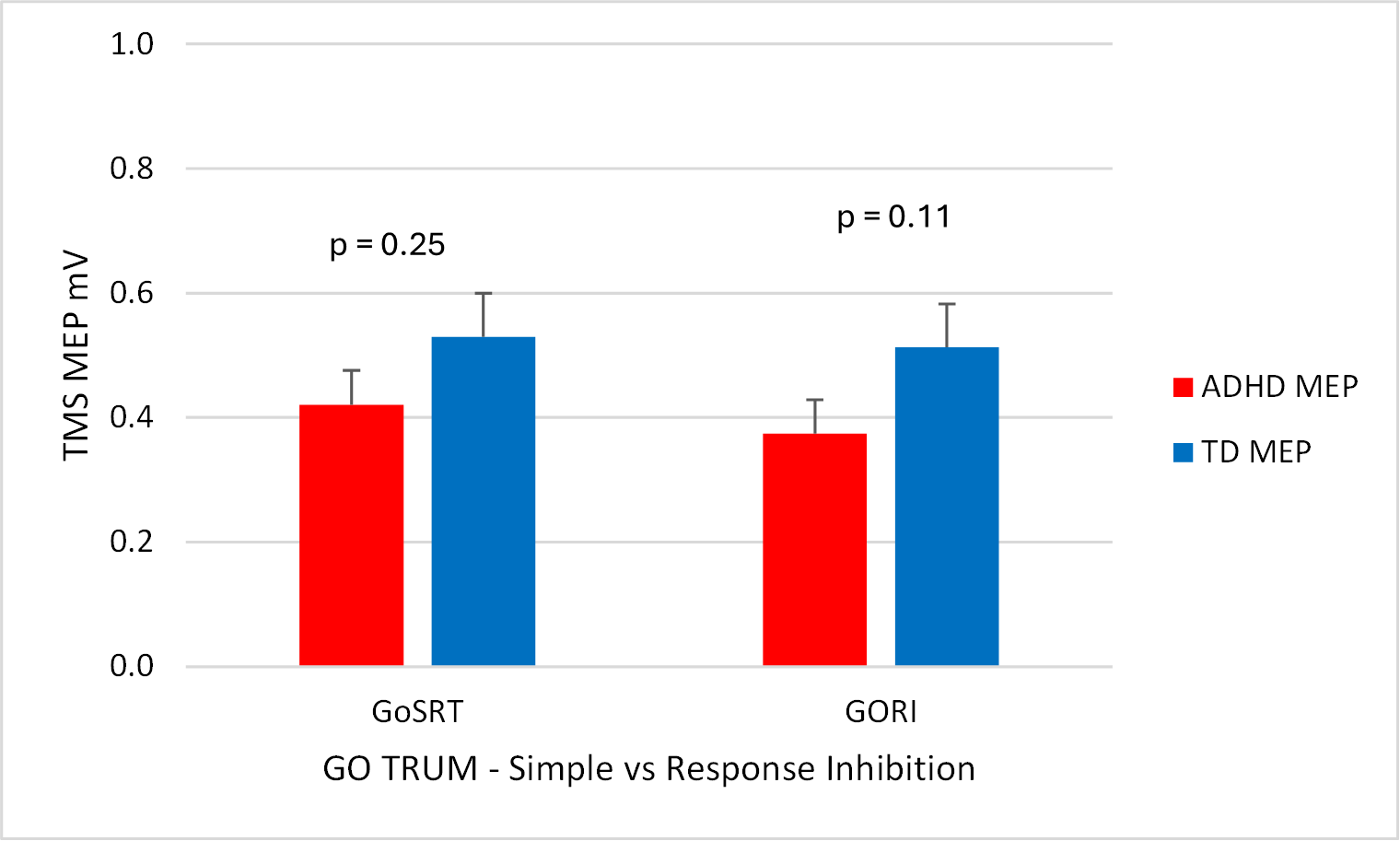


**Supplemental Figure 3. TRUM GO in Simple Reaction Time vs. Response Inhibition experiments**

We compared GO-TRUM between the simple reaction time (GO-Only GO trials) and response inhibition task to determine whether GO-TRUM reflects the presence or absence of an instruction to “possibly inhibit”. STOP trials were excluded from this model. Post hoc diagnosis effects were not significant.

***Solution***

***Type III***

***Post hoc***

1. **Dimensional exploratory analyses for TRUM, all trial types, ADHD-RS and SSRT**

In the primary TRUM analysis for response inhibition, diagnosis is categorical (ADHD children vs TD controls) (MEP = Dx + Trialtype + Diagnosis*Trialtype) (see methods). In the following analysis of response inhibition TRUM, diagnosis is removed from this full model and replaced by parent-rated ADHD symptom severity (Models S4 A, B, and C: higher numbers, worse symptoms) and by SSRT (Model S4 D; longer times, less efficient response inhibition).

**Supplemental Table 4A. MEP = ADHD-RS(inattentive) + Trialtype**

**Type III regression output**

| **Effect** | **NumDF** | **DenDF** | **F** | **p** |
| --- | --- | --- | --- | --- |
| TrialType | 2 | 9389.074 | 98.427 | 0.000 |
| ADHD-RS Inattentive | 1 | 65.077 | 1.913 | 0.171 |
| Trialtype * ADHD-RS-I | 2 | 9389.026 | 10.750 | 0.000 |

**Supplemental Figure 4A. TRUM by task (Trialtype) and ADHD-RS-I**


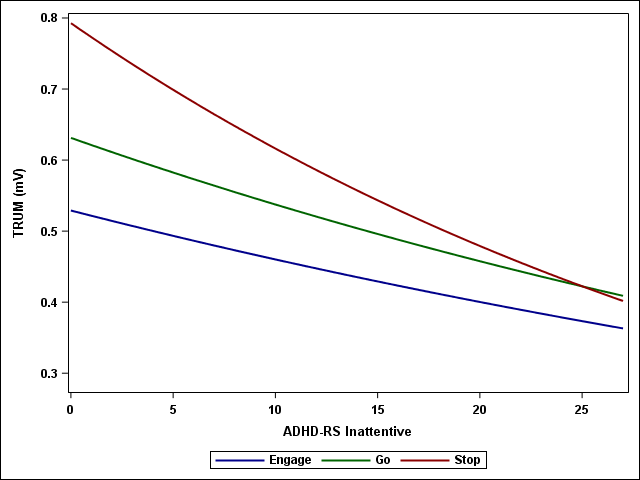


Regression lines generated from mixed model. X axis is ADHD-RS inattentive subscale, maximum score 27. Y axis is TRUM in millivolts. TRUM increases with task “cognitive load”: largest for stop, then preparing to go, then engaging at the onset of the task. Among more inattentive children (right), this task-load difference in TRUM tends to diminish.

**Supplemental Table 4B. MEP = ADHD-RS(hyper/impulsive) + Trialtype**

**Type III regression output**

| **Effect** | **NumDF** | **DenDF** | **F** | **p** |
| --- | --- | --- | --- | --- |
| TrialType | 2 | 9389.151 | 125.018 | 0.000 |
| ADHD-RS Hyper/Impulsive | 1 | 65.075 | 2.722 | 0.104 |
| Trialtype * ADHD-RS-HI | 2 | 9389.028 | 17.816 | 0.000 |

**Supplemental Figure 4B. TRUM by task (Trialtype) and ADHD-RS-H/I**


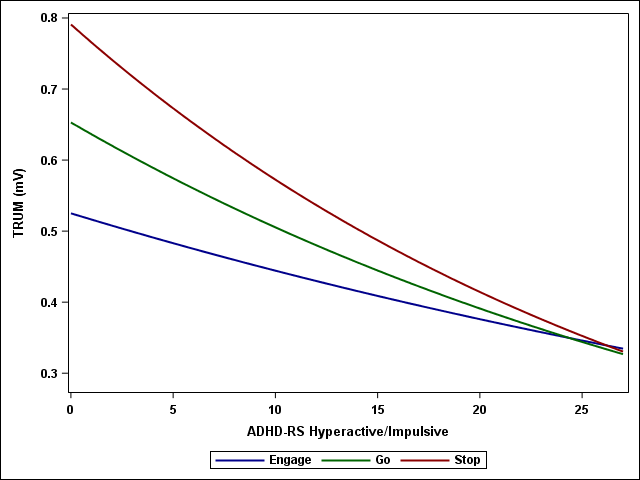


Regression lines generated from mixed model. X axis is ADHD-RS hyperactive/impulsive subscale, maximum score 27. Y axis is TRUM in millivolts. TRUM increases with task “cognitive load”: largest for stop, then preparing to go, then engaging at the onset of the task. Among more Hyperactive/Impulsive children (right), this task-load difference in TRUM tends to diminish.

**Supplemental Table 4C. MEP = ADHD-RS(total) + Trialtype**

**Type III regression output**

| **Effect** | **NumDF** | **DenDF** | **F** | **p** |
| --- | --- | --- | --- | --- |
| TrialType | 2 | 9389.103 | 110.103 | 0.000 |
| ADHD-RS Total | 1 | 65.077 | 2.448 | 0.123 |
| Trialtype * ADHD-RS-T | 2 | 9389.021 | 13.967 | 0.000 |

**Supplemental Figure 4C. TRUM by task (Trialtype) and ADHD-RS-Total**


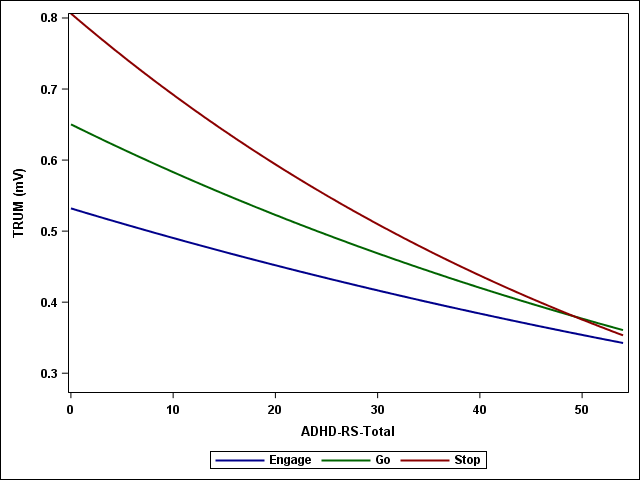


Regression lines generated from mixed model. X axis is ADHD-RS total scale (combines inattentive with hyper/impulsive), maximum score 54. Y axis is TRUM in millivolts. TRUM increases with task “cognitive load”: largest for stop, then preparing to go, then engaging at the onset of the task. Among more severely affected children (right), this task-load difference in TRUM tends to diminish.

**Supplemental Table 4D. MEP = SSRT + TrialType**

**Type III regression output**

| **Effect** | **NumDF** | **DenDF** | **F** | **p** |
| --- | --- | --- | --- | --- |
| TrialType | 2 | 10300.084 | 52.799 | 0.000 |
| SSRT | 1 | 73.092 | 0.041 | 0.839 |
| Trialtype * SSRT | 2 | 10300.085 | 25.288 | 0.000 |

**Supplemental Figure 4D. TRUM by task (Trialtype) and SSRT**


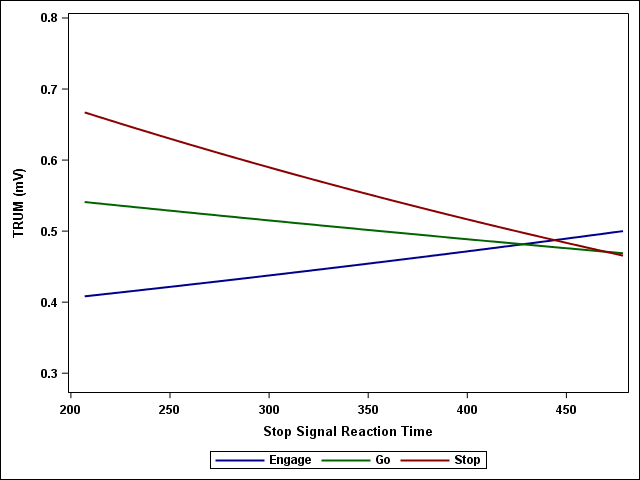


Regression lines generated from mixed model. X axis is Stop Signal Reaction Time (SSRT, in millisecond), longer indicates worse response inhibition. Y axis is TRUM in millivolts. TRUM increases with task “cognitive load”: largest for stop, then preparing to go, then engaging at the onset of the task. Among more children with longer SSRTs (right), this task-load difference in TRUM tends to diminish.

**TRUM REGRESSION OUTPUT**

**TRUM – Simple Reaction Time**

1. Is pre-movement artifact different in ADHD? NO EVIDENCE

1. Does pre-movement motor cortex activation depend on timing of pulse versus time of finger lift? NO EVIDENCE p for movetime = 0.16

1. Does Movetime effect on MEPs depend on diagnosis? APPROACHES SIGNIFICANCE – more effect within TD than ADHD

1. Is TRUM different for START(250) vs. GO(650)? YES

1. Does the effect of START(250) vs. GO(650) depend on diagnosis? – NO EVIDENCE

**TRUM – Response inhibition**

1. Is TRUM (MEP) different for START(250) vs. GO(650) vs. STOP? YES

1. Does the effect of task depend on diagnosis? YES

1. Post Hoc 1 Does STOP-TRUM depend on diagnosis? YES
2. Post Hoc 2 Does GO-TRUM depend on diagnosis? NO
3. Post Hoc 3 Does START-TRUM depend on diagnosis? NO
